# Bioactivity of selenium nanoparticles biosynthesized by crude phycocyanin extract of *Leptolyngbya* sp. SSI24 cultivated on recycled filter cake wastes from sugar-industry

**DOI:** 10.1186/s12934-024-02482-2

**Published:** 2024-07-26

**Authors:** Sara Saad, Amr Mohamed Abdelghany, Ghada Samir Abou-ElWafa, Heshmat Soliman Aldesuquy, Eladl Eltanahy

**Affiliations:** 1 Botany Department, Faculty of Science, Mansoura University, Mansoura, 35516 Egypt; 2 Spectroscopy Department, Physics Research Institute, National Research Center, Giza, 12311 Egypt

**Keywords:** Beet filter cake, Cyanobacteria, Phycocyanin, Selenium nanoparticles

## Abstract

**Background:**

Beet filter cake (BFC) is a food-grade solid waste produced by the sugar industry, constituting a permanent source of pollution. Cyanobacteria are considered a sustainable resource for various bioactive compounds such as phycocyanin pigment with valuable applications. This study aimed to use beet filter cake extract (BFCE) as an alternative medium for the economic cultivation of cyanobacterium *Leptolyngbya* sp. SSI24 PP723083, then biorefined the bioactive component such as phycocyanin pigment that could be used in the production of selenium nanoparticles.

**Results:**

The results of the batch experiment displayed that the highest protein content was in BG11medium (47.9%); however, the maximum carbohydrate and lipid content were in 25% BFCE (15.25 and 10.23%, respectively). In addition, 75% BFCE medium stimulated the phycocyanin content (25.29 mg/g) with an insignificant variation compared to BG11 (22.8 mg/g). Moreover, crude phycocyanin extract from *Leptolyngbya* sp SSI24 cultivated on BG11 and 75% BFCE successfully produced spherical-shaped selenium nanoparticles (Se-NPs) with mean sizes of 95 and 96 nm in both extracts, respectively. Moreover, XRD results demonstrated that the biosynthesized Se-NPs have a crystalline nature. In addition, the Zeta potential of the biosynthesized Se-NPs equals − 17 mV and − 15.03 mV in the control and 75% BFCE treatment, respectively, indicating their stability. The biosynthesized Se-NPs exhibited higher effectiveness against Gram-positive bacteria than Gram-negative bacteria. Moreover, the biosynthesized Se-NPs from BG11 had higher antioxidant activity with IC_50_ of 60 ± 0.7 compared to 75% BFCE medium. Further, Se-NPs biosynthesized from phycocyanin extracted from *Leptolyngbya* sp cultivated on 75% BFCE exhibited strong anticancer activity with IC_50_ of 17.31 ± 0.63 µg/ml against the human breast cancer cell line.

**Conclusions:**

The BFCE-supplemented medium can be used for the cultivation of cyanobacterial strain for the phycocyanin accumulation that is used for the green synthesis of selenium nanoparticles that have biological applications.

**Graphical Abstract:**

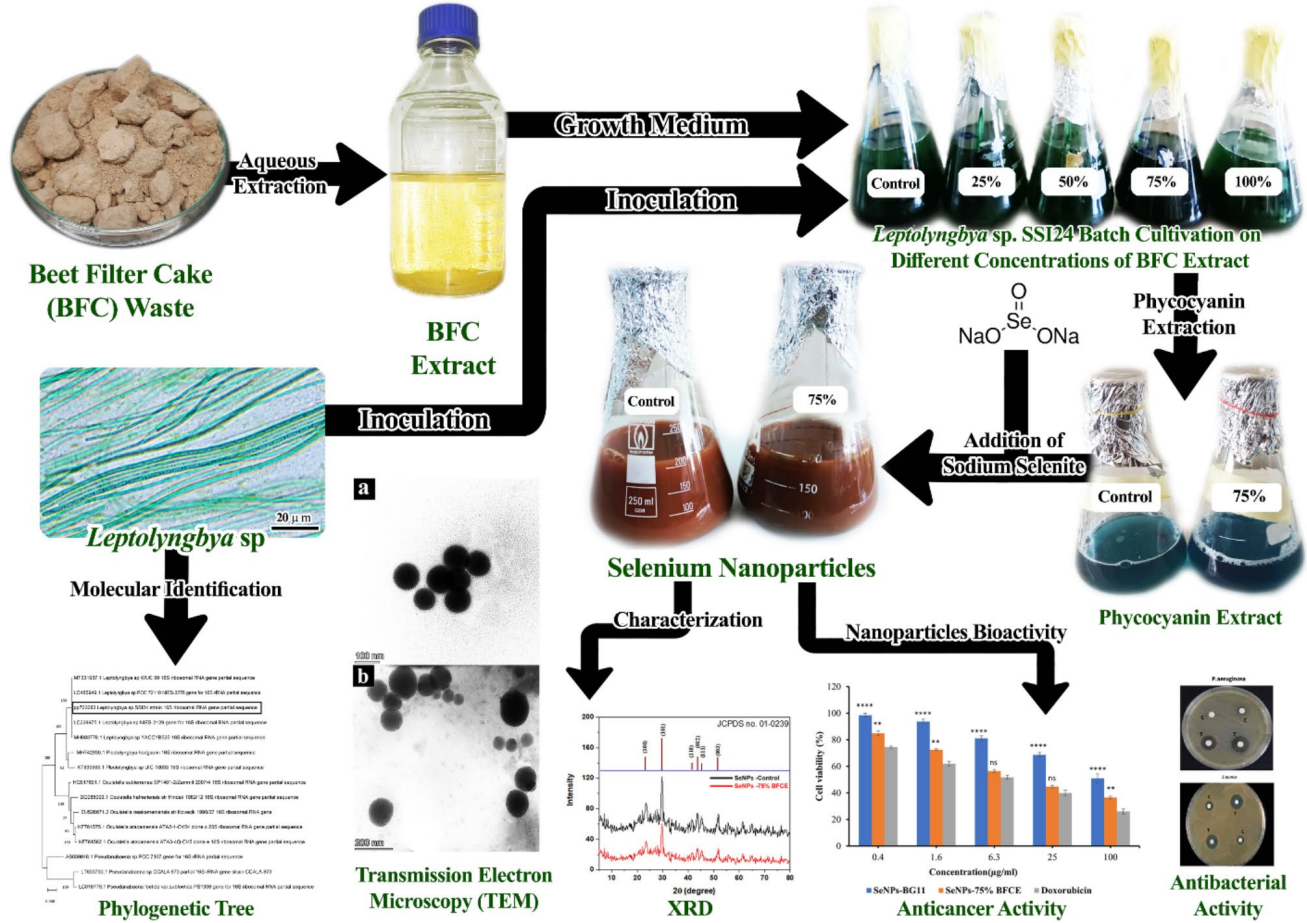

## Background

The use of waste materials of food grade as substrates for obtaining biomass containing promising bioactive components is highly preferable as it is an eco-friendly technology and simultaneously reduces the cost related to both waste control and microalgae production [1].

Beet filter cake (BFC) is a food-grade solid waste produced from the sugar industry, constituting a permanent source of solid pollution. The BFC is rich in calcium carbonates, organic matter, and some minerals. Thus, this waste can be utilized as an alternative medium for cultivating valuable cyanobacteria with promising high-value products [2].

In fact, cyanobacteria are interesting microorganisms due to their ability to manufacture several bioactive metabolites such as valuable pigments, proteins, polysaccharides, and vitamins with critical biotechnological applications [3]. *Leptolyngbya* sp. is among filamentous unbranched non heterocystous cyanobacterial strain that has the potential to accumulate a high value phycocyanin under desert conditions [4].

Phycocyanin is a valuable water-soluble phycobiliprotein produced within the cyanobacterial cell [5, 6]. It is well known that phycocyanin pigment is utilized as a colorant in food processing or natural cosmetic dyes. Also, it has therapeutic activities such as antioxidant, anti-inflammatory, neuroprotective, and anticancer [7, 8]. Furthermore, recent minor studies used phycocyanin extracted from cyanobacteria in nanobiotechnology [9, 10].

Recently, selenium nanoparticles (Se-NPs) have obtained more attention due to its potential utility in various applications [11]. Selenium nanoparticles are typically synthesized by conventional physical and chemical techniques, which produce expensive, unsafe nanoparticles that limit its application in the medical field. Therefore, the biological (green) method has replaced the conventional techniques, satisfying the increasing demand for safe, inexpensive, and eco-friendly nanoparticles [12]. Various organisms are incorporated into the biosynthesis of Se-NPs, including fungi, bacteria, cyanobacteria, and higher plants [10, 13–15]. Selenium nanoparticles can be produced intracellular or extracellular through extracted biomolecules, namely, protein, polysaccharides, pigments, phenols, sugars, and flavonoids acting as reducing agents as well as capping and stabilizer agents in the synthesis of selenium nanoparticles [16].

Afzal B, Yasin D, Husain S, Zaki A, Srivastava P, Kumar R and Fatma T [10] tested the ability of cyanobacterial crude extract to manufacture selenium nanoparticles, and Yang F, et al. [17] focused on the usage of *Spirulina platensis* polysaccharides for synthesizing the Se-NPs and their anticancer activity.

Therefore, this study investigated the feasibility of cyanobacterial isolate growing on the BFCE as an alternative economic cultivation medium, in addition to studying the influence of this medium on chemical composition, especially the phycocyanin content. Finally, screening the extracted phycocyanin in the biological production of selenium nanoparticles and studying their antibacterial, antioxidant, and anticancer activity.

## Materials and methods

### Cyanobacteria isolation and culture conditions

Water samples were collected from the River Nile in Minya city (Egypt). Then, isolation and purification of cyanobacterial isolate were performed on BG11 medium. The axenic filaments were shifted to a sterile 500 mL flask containing BG11 medium. The cultures were grown under a fluorescent lamp at a light intensity of 3600 lx for 16 h light/ 8 h dark cycle at 26 ± 2 °C, under constant aeration (The pumped air was sterilized first by passing through bacterial air filters of 0.22µ pore diameter). This culture was maintained for 7–10 days.

### Cyanobacterium isolate identification

The identification of cyanobacterium was performed based on both its morphological characteristics using light microscope and molecular identification using 16s rRNA gene sequencing. DNA extraction was done according to Cheng H-R, et al. [18]. A cyanobacterial 16 S rRNA gene fragment was amplified using the universal primers 27 F (5′- AGAGTTTGATCCTGGCTAG − 3′) and 1492R (5′- GGTTACCTTGTTACGACTT − 3′). The amplification was performed using Thermal cycle PCR. The thermocycling conditions implied an initial denaturation cycle for 5 min at 94ºC, followed by 40 cycles. Each cycle consisted of a denaturation step at 94ºC for 30 s., an annealing step at 45ºC for 30 s., and an elongation step at 72ºC for 1 min. The primer extension segment was extended to 7 min at 72ºC in the final cycle. Amplified products for all PCR were purified using EZ-10 spin column PCR product purification. The sequencing of the PCR product was carried out in an automated DNA sequencer ABI PRISM 3730XL Analyzer by Sanger sequencing method with the help of Big Dye TM Terminator Cycle Sequencing Kits. Sequence similarity was analyzed using a BLAST search after multiple alignments with sequences of closely related species. The generated sequence was deposited in the Gene Bank. The phylogenetic tree was constructed using the Maximum Likelihood method using MEGA X [19].

### Collection, preparation, and analysis of BFC

The BFC was collected from Dakahlia Sugar Factory, Dakahlia province, Egypt. The preparation and chemical composition analysis of BFCE was carried out according to Saad S, et al. [20] and compared to BG11 medium composition as shown in Table 1.

**Table 1:** Comparison between the composition of BG11 and BFCE

Component	Concentration in ppm	
	BG11	BFCE
Macronutrients		
Nitrogen (N)	247	2.77
Phosphorus (P)	7.11	50.1
Potassium (K)	17.9	34.87
Calcium (Ca)	9.81	565.7
Magnesium (Mg)	11.66	55.97
Sodium (Na)	414.5	391.06
Micronutrients		
Iron (Fe)	1.28	22.84
Boron (B)	499.8	0.97
Manganese (Mn)	502.4	0.99
Zinc (Zn)	50.47	1.41
Molybdenum (Mo)	155.09	ND
Cobalt (Co)	12.38	0.15
Copper (Cu)	56.49	ND

*ND = Not detected

### Batch cultivation of cyanobacterium on BFCE

The BFCE-supplemented cultures were prepared by adding different volumes of beet filter cake extract (BFCE) to the BG11 medium, while BG11 only served as the control treatment. The constituents of BG11 were eliminated stepwise and substituted with an increasing percentage volume of BFCE (BG11, 25%, 50%, 75%, and 100% BFCE). The experiment was conducted in a replicate for each treatment. All treatments proceeded with a total volume of 400 ml in a 500 ml Erlenmeyer flask, then inoculated with 10% (v/v) exponentially growing culture (O.D = 0.8 at 680 nm). At the same time, the incubation temperature was 26 ± 2°C, 50 µmol/s/m^2^ illuminations, 16:8 h light: dark cycle, and continuous bubbling with sterile airflow for seven days.

### Analytic methods

#### Growth parameters assay

For the photosynthetic performance assessment, chlorophyll fluorescence was measured daily in dark-adapted cultures of cyanobacterium isolate grown in BG11 and BFCE using a pulse amplitude modulation fluorometer (PAM) (AquaPen AP 110-C). The maximum photochemical efficiency of photosystem II (Fv/Fm) was measured [21]. The culture optical density was also measured daily using a spectrophotometer at a wavelength of 680 nm [22]. For dry weight estimation, the culture of cyanobacterium was harvested by centrifugation at 6000 rpm for 10 min and washed with distilled water to remove any salt remaining, then centrifuged again under the same conditions. The pellet of the cyanobacterium isolate was dried in a lyophilizer for 48 h at -50 °C and weighed [23].

#### Photosynthetic pigments

##### Chlorophylls and carotenoids

The pigment fraction (chlorophyll-a, chlorophyll-b, and carotenoids) was extracted by a known volume of 80% acetone and then measured spectrophotometrically at wavelengths 664, 647, 630, and 452 nm, respectively. The pigment concentrations were calculated by Jeffrey St, et al. [24] equations.

##### Phycocyanin estimation

Phycocyanin was extracted from cyanobacterium isolate using 0.056 g of lyophilized cyanobacterium isolate using a 10 ml phosphate buffer 0.1 M (PH = 7.2),, followed by freezing-thawing cycles and homogenization two times. The crude phycocyanin extract was obtained by centrifugation at 4000 rpm for 10 min. The absorbance of the supernatant was determined at wavelengths of 620 and 650 nm, using phosphate buffer as a blank. Phycocyanin concentration (W/V) and the purity of phycocyanin extract were calculated by the following equations of Bennett A, et al. [25].

$${\rm{PC}}\,\left( {{\rm{mg}}/{\rm{ml}}} \right)\, = \,{{\rm{A}}_{620}} - \left( {0.472*{{\rm{A}}_{650}}} \right)/5.34$$

$${\rm{Phycocyanin}}\,{\rm{Purity}} = \,{{\rm{A}}_{620}}/{{\rm{A}}_{280}}$$

The phycocyanin yield was determined according to the equation of Silveira ST, et al. [26].

$${\rm{PC}}\,\left( {{\rm{mg}}/{\rm{g}}} \right)\, = \,{\rm{PC }}\left( {{\rm{mg}}/{\rm{ml}}} \right)\, \times {\rm{SV}}/\,{\rm{DW}}\left( {\rm{g}} \right)$$

Where SV is the solvent volume and DW is the dry biomass (g).

#### Biochemical characteristic determination

Protein content was determined according to Lowry’s method [27]. The protein estimation was carried out by cell lysis using bovine serum albumin as a standard. Total carbohydrates were determined according to the phenol sulfuric acid method described by Dubois M, et al. [28], and the concentration of carbohydrates was calculated from the standard curve of glucose. Lipid concentration was estimated using the sulfo-phospho-vanillin method described by Byreddy AR, et al. [29], and the lipid content was calculated using a standard curve for cholesterol.

### Biosynthesis of selenium nanoparticles using crude phycocyanin extract from the cyanobacterium isolate

Biosynthesis of selenium nanoparticles using crude extract of phycocyanin from cyanobacterium isolate cultivated on both BG11 and 75% BFCE supplemented medium. Selenium nanoparticles were synthesized by carefully adding 0.0526 g of pentahydrate Na_2_SeO_3_ powder to 100 ml phycocyanin extract to make a final concentration of 2 mM. The reaction mixture was adjusted to obtain the best yield at 30 °C, pH = 7.2 after salt addition under shaking at 120 rpm in dark conditions to prevent photooxidation for 48 h until the color change occurred from blue to red orange. Sodium selenite solution (2 mM) without phycocyanin extract was also kept under identical conditions as a control. The prepared nanoparticles were immediately used after the preparation process without evidence for agglomeration. The synthesized Se-NPs were then collected by centrifuging at 12,000 rpm for 20 min. The pellet of Se-NPs obtained was washed thrice with double distilled water and acetone to remove unreduced salts and unused capping agents. Afterward, the washed Se-NPs were dried overnight at room temperature to obtain purified Se-NPs for further characterization.

#### Characterization of synthesized selenium nanoparticles

After completion of the reaction, 2 ml of the biogenic Se-NPs were centrifuged at 12,000 rpm for 20 min, the supernatant was removed, and the pellet was resuspended with deionized water for evaluating the absorption peak by UV–Vis Spectrophotometer across the wavelength range of 190.00 nm to 1100.0 nm at a resolution of 1 nm. The confirmation of selenium nanoparticles synthesis was done by various techniques like Transmission Electron Microscopy (**JEOL JEM-2100**,** U.S.A**) using a carbon-coated grid **(Type G 200**,** 3.05µ diameter**,** TAAP**,** U.S.A)** according to the method of [30] that analyzed the size and morphology of biologically synthesized Se-NPs [31], The X-ray diffraction (**XRD**,** Bruker co.**,** D8 Discover**,** Germany**) using Cu Kα (1.54Å) radiation with the X-ray generator operating at 40 kV and 40 mA to determine the crystalline nature of the selenium nanoparticles and the crystallite size was calculated from XRD data based on Scherrer’s equation [32].

$$\:D=\:\frac{K\:\lambda\:}{\beta\:\:cos\theta\:}$$

Where:

D is the mean crystallite size, K is the shape factor usually used as 0.9 for spherical particles, λ is the wavelength of X-ray used, β is the full width at half maximum (FWHM) of the diffraction peak (in radians), and θ is the Bragg diffraction angle.

Dynamic Light Scattering (DLS) Spectroscopy (**Malvern Zeta size Nano-zs90**,** U.S.A**) was used for determination of the zeta potential of synthesized and their size (hydrodynamic diameter). Furthermore, Fourier Transform Infra-Red (FTIR) (**BRUKER ALPHA** II, **Germany**) in the spectral range 400–4000 cm^− 1^ and spectral resolution 2 cm^− 1^ was carried out for the determination of the functional groups responsible for the biosynthesis and stabilization of selenium nanoparticles.

#### Application of biosynthesized selenium nanoparticles

##### Antibacterial activity of Se-NPs

The antibacterial activity of Se-NPs against *Staphylococcus aureus*, *Staphylococcus Epidermidis*, *Staphylococcus pneumoniae*,* Klebsiella pneumoniae*, and *Pseudomonas aeruginosa* was determined by disc-diffusion method as follows: commercially prepared sterile discs of 6 mm diameter were infused with 100 µl of prepared Se-NPs, dried, and deposited aseptically onto plates inoculated with 1 ml of tested bacteria, chloramphenicol 30 µg/ml was used as a positive control. After incubating at 37 °C for 24 h, the inhibition zone was determined in mm with a ruler.

##### Antioxidant activity of the biosynthesized Se-NPs using DPPH radical scavenging

Synthesized Se-NPs were evaluated for their antioxidant activity using DPPH radical scavenging. This method depends on the decolorizing of DPPH in the presence of antioxidants. DPPH reagent was prepared as follows, 0.004 g of DPPH was dissolved in 100 ml methanol reagent, then a specific volume of Se-NPs at various concentrations (50–350 µg/ml), and DPPH solution was thoroughly mixed, and then incubated in the dark for 30 min. Finally, a UV-visible spectrophotometer was used to estimate the absorbance at 517 nm. Synthetic antioxidant such as Torlox was used to compare the antioxidants of the samples.

The degree of scavenging activity was calculated by the following equation:

$$\eqalign{{\rm{DPPH}}\, - {\rm{radicle}}\,{\rm{scavenging}}\,\left( \% \right)\, = \, & \cr & \left( {\left( {{{\rm{A}}_{\rm{c}}}\,-\,{{\rm{A}}_{\rm{s}}}} \right)/{{\rm{A}}_{\rm{c}}}} \right)\, \times \,100. \cr}$$

Where A_c_ is the absorbance of the control and A_s_ is the absorbance of the sample.

The required concentration of the test samples for inhibiting 50% of DPPH (IC_50_) was calculated by linear regression. The lower the IC_50_ value, the more potent the substance at scavenging DPPH.

##### Anticancer activity of Se-NPs by MTT assay

The inhibitory effect of the biosynthesized Se-NPs against the human cancer cell line, breast cancer cell line (MCF-7) was evaluated by 3-(4,5-dimethylthiazol-2-yl)-2,5-diphenyl tetrazolium bromide (MTT), in which yellow MTT reagent is converted to purple formazan in the mitochondria of living cells. As this reduction occurs solely when the mitochondrial reductase enzymes are in operation, the conversion is directly correlated with the quantity of viable cells. The cell line was grown in Minimum Essential Medium (MEM) EARLES medium (Sigma Aldrich, USA) supplemented with 10% fetal calf serum (FCS) (GEPCO, USA), 100 µg/ml penicillin and 10 µg/ml streptomycin. The 96-well plate was seeded with 10^3^ cells/well and incubated overnight at 37 °C. Then, different concentrations of Se-NPs were added, and the plates were re-incubated for 48 h. The MTT reagent (25 µl) was added and incubated for 2 h, and the purple color of the developed formazan complex was measured at 570 nm [33]. The percentage of cell survival was calculated using the following formula:

$${\rm{Cell}}\,{\rm{survival}}\,\left( \% \right)\, = \,\left( {{\rm{A}}\,{\rm{sample}}\,-\,{\rm{A}}\,{\rm{blank}}} \right)/\,\left( {{\rm{A}}\,{\rm{control}}\, - {\rm{A}}\,{\rm{blank}}} \right)\, \times 100.$$

Where A control is the absorbance of the control reaction without drug, A blank is the absorbance of the blank, and A sample is the absorbance in the presence of the sample. Each test was done in triplicate, and the IC_50_ value was determined, which was expressed by the number of Se-NPs reducing the growth of 50% of the initial number of cells normalizing to negative controls (without drug).

### Statistical analysis

The mean and standard error (mean ± SE) were calculated from the triplicate experimentations. The one-way ANOVA was applied to identify the significant differences between treatments using Duncan’s test at a significant level of *p* ≤ 0.05. Moreover, Two-way ANOVA with Tukey’s multiple comparisons test to compare the treatment in anticancer activity test in comparing with reference, ****p-level < 0.0001, **p-level < 0.01, and ns p-level > 0.05.

## Results

### Morphological and molecular identification

**Fig. 1:**
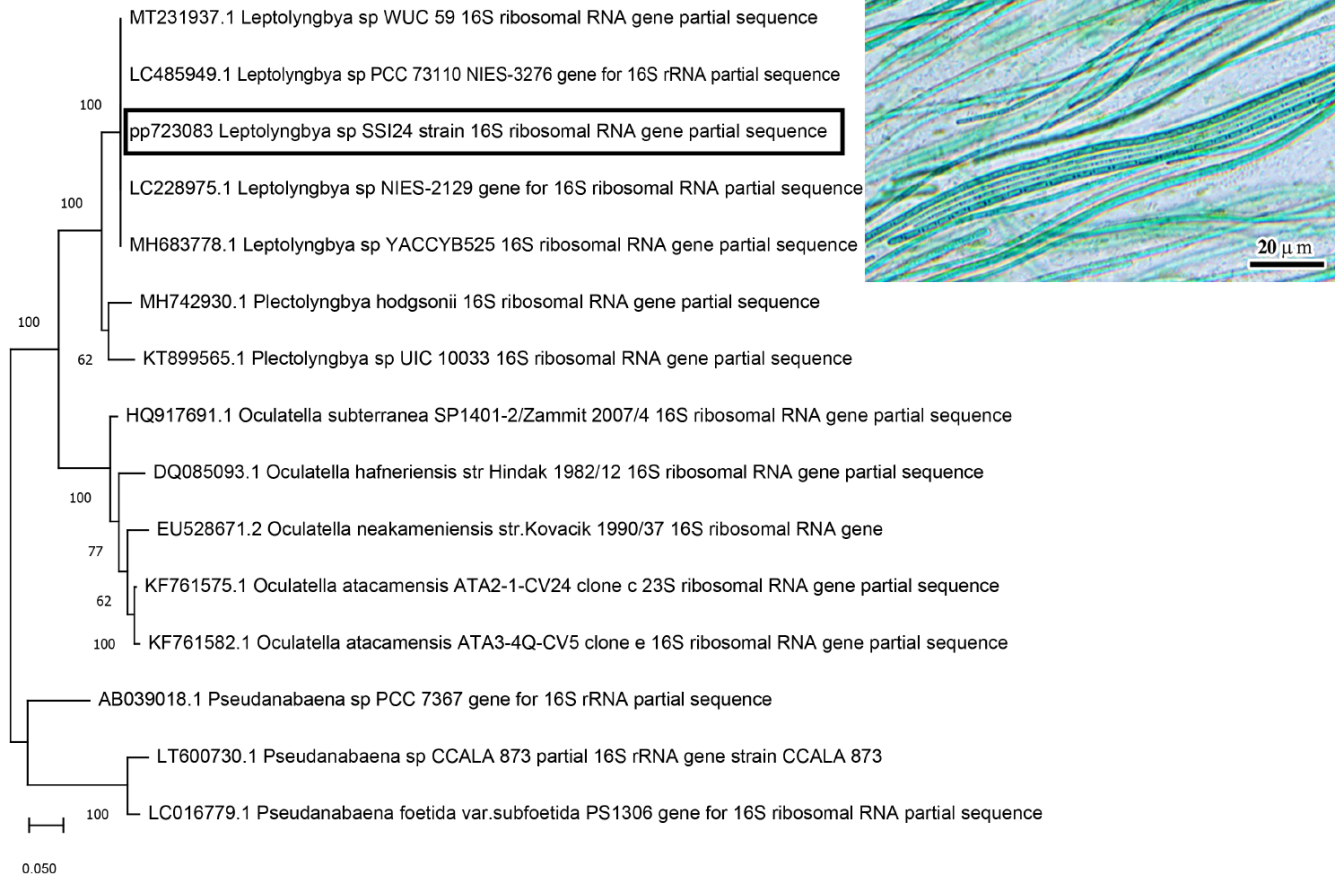
Phylogenetic tree showing the relationship between *Leptolyngbya* sp. SSI24 with closely related cyanobacterial strain based on partial 16SrRNA gene sequence

The cyanobacterial strain was identified under light microscope for its morphological characteristics, moreover identification based on the partial 16 S rRNA gene sequence. The isolate was unbranched, densely packed filament, frequently spirally or densely snake- like. The trichome was long having a firm sheath. Moreover, the filaments were motile, and the cells were longer than wide with one or two granules on either side of cross-walls Sequencing results displayed that this isolate was closely related to *Leptolyngbya* sp. WUC 59 with accession number (MT231937). Therefore, this strain was identified as *Leptolyngbya* sp. SSI24 and has been deposited in Gene Bank database with an accession number of PP723083 (Fig. 1).

### Batch cultivation of *Leptolyngbya* sp. on BFCE

#### Photosynthetic activity

**Fig. 2:**
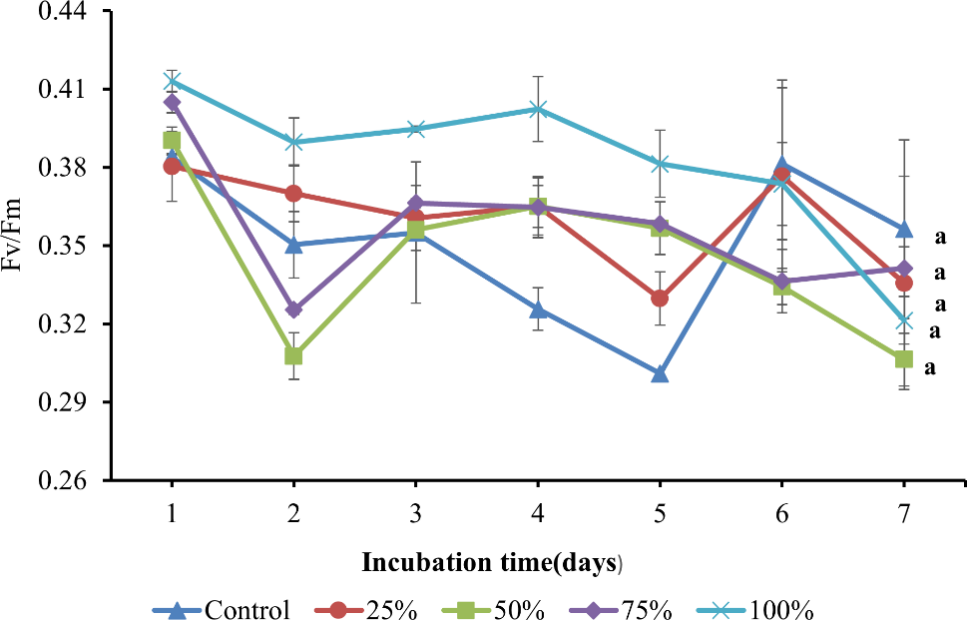
Optimal quantum yield (Fv/Fm) of *Leptolyngbya* sp. ± SE cultivated in the BG11 and BFCE treatments

As shown in Fig. 2, the photosynthetic activity was reflected by the maximum optical quantum yield (Fv/Fm). Initially, the Fv/Fm values in both BG11 and BFCE medium were almost similar, ranging from 0.38 ± 0.0005 to 0.41 ± 0.0041. On the second day of the experiment, the Fv/Fm values of all treatments were reduced, and then adaptation patterns occurred with an increase in Fv/Fm values as the *Leptolyngbya* sp took time to adapt to grow on BFCE. Moreover, on the 7th day, all treatments’ Fv/Fm values decreased again. In conclusion, Fv/Fm was affected by a 7% difference between all treatments during the experiment but all the treatments’ differences were not significant.

#### Evaluation of growth by optical density

**Fig. 3:**
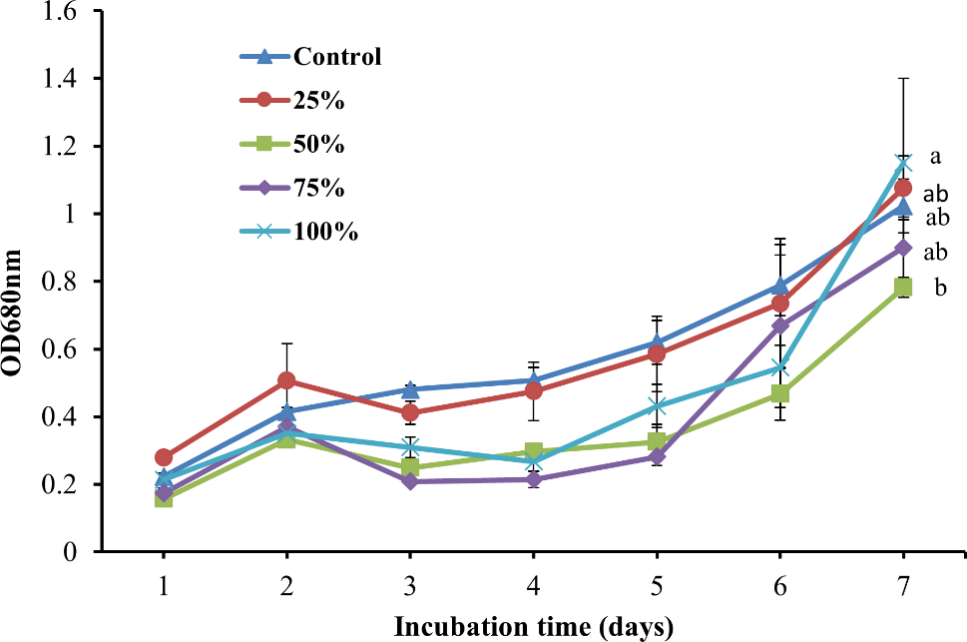
The optical density of *Leptolyngbya* sp. ± SE cultivated in the BG11 and BFCE treatments

The optical density of *Leptolyngbya* sp. as an indirect growth parameter indicated an increase in all investigated treatments with the same trend, all treatments increased in the 2nd day of cultivation, but a sudden decrease occurred on 3rd day, then from the 5th day, the growth increase until reached to maximum at the end of cultivation period (Fig. 3). Furthermore, there was a non-significance difference between the growth at 100% BFCE (0.21 ± 0.001 to 1.15 ± 0.25) and the BG11 medium (0.22 ± 0.01to 1.022 ± 0.07) at *p* ≤ 0.05, indicating its acclimation to the BFCE medium.

#### Estimation of dry-weight biomass

**Fig. 4:**
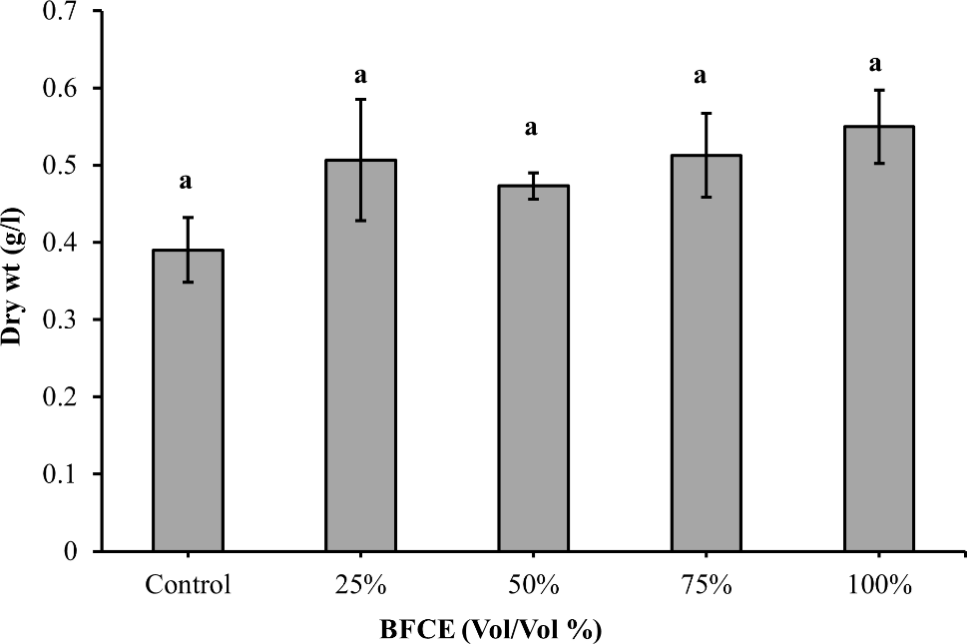
Dry weight of *Leptolyngbya* sp. ± SE cultivated in the BG11 and BFCE treatments

As shown in Fig. 4, all media supplemented with BFCE showed considerably higher dry biomass, ranging between 0.47 ± 0.01 and 0.54 ± 0.04 g/l. The minimum value was observed at BG11 (0.38 ± 0.042 g/l), while the maximum biomass was in 100% BFCE (0.54 ± 0.047 g/l), and the dry weight of 100% BFCE increased by 42% over BG11. However, the variation in dry cell weight in BG11 was a non-significant decrease (*p* ≤ 0.05) compared to the BFCE treatments.

#### Photosynthetic pigment estimation

##### Chlorophyll a, b, and carotenoids

**Fig. 5:**
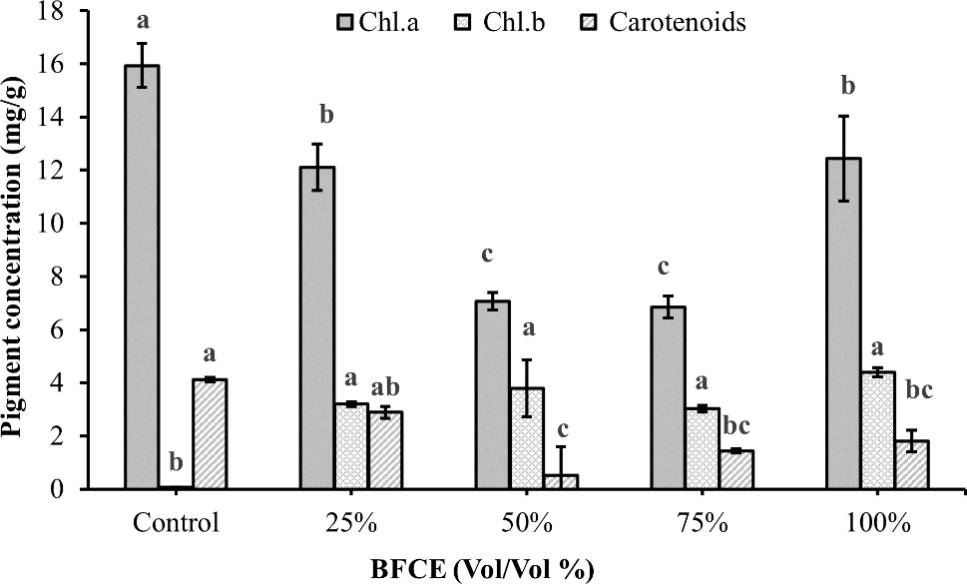
Photosynthetic pigments of *Leptolyngbya* sp. ± SE cultivated in the BG11 and BFCE treatments

As illustrated in Fig. 5, the maximum chlorophyll-a concentration (15.93 ± 0.8 mg/g) was observed in the control medium (BG11), while the minimum value (6.85 ± 0.4 mg/g) was in 75% BFCE. The variation in pigment concentrations was significantly decreased (*p* ≤ 0.05) in different concentrations of BFCE compared to the BG11. In contrast, the chlorophyll b concentrations increased in all concentrations of BFCE compared to the control medium (BG11). Furthermore, the carotenoid content significantly decreased (*p* ≤ 0.05) in the BFCE concentrations compared to the BG11 medium as a control.

##### Phycocyanin determination

**Fig. 6:**
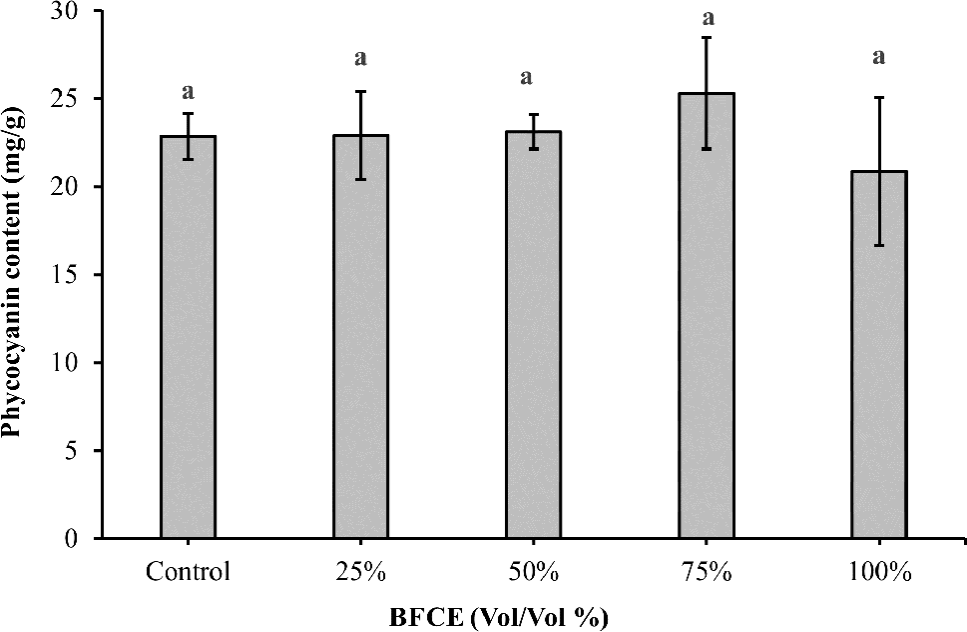
Phycocyanin content of *Leptolyngbya* sp. ± SE cultivated in the BG11 and BFCE treatments

According to Fig. 6, the phycocyanin content in the control medium was 22.8 ± 1.27 mg/g. Furthermore, the supplementation with different BFCE concentrations had a positive effect on phycocyanin content up to 75% BFCE (25.29 ± 3.17 mg/g). On the other hand, the variation in phycocyanin content between the control and BFCE treatments was insignificant (*p* ≤ 0.05). Moreover, the phycocyanin purity was summarized in Table 2.

**Table 2:** The purity of phycocyanin of *Leptolyngbya* sp. Cultivated in the BG11 and BFCE treatments

Treatment	OD620	OD652	OD280	DF620	DF 280	Purity A620/A280 ratio	Phycocyanin Concentrations (mg/ml)
Control	0.745	0.5115	0.227	1.5	1	4.92	0.138
25% BFCE	0.6435	0.7005	0.166	2	1	7.73	0.114
50% BFCE	0.6895	0.9865	0.164	3	1	12.6	0.122
75% BFCE	0.624	0.8225	0.162	3	1	11.5	0.129
100% BFCE	0.529	0.6975	0.145	3	1	10.9	0.109

#### Estimation of biochemical composition

##### Protein determination

**Fig. 7:**
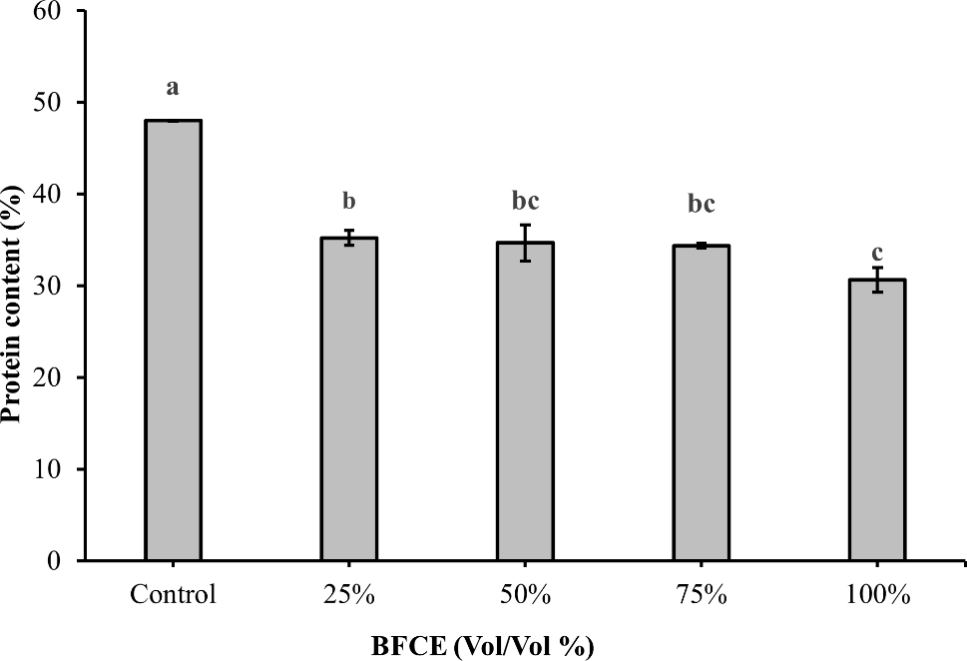
Protein content of *Leptolyngbya* sp. ± SE cultivated in the BG11 and BFCE treatments

The supplementation with BFCE to the BG11 medium significantly decreased (*p* ≤ 0.05) the protein content compared to the control medium (BG11 only) (Fig. 7). The maximum protein content was 47.99 ± 0.05% at BG11 followed by 25% (35 ± 0.8%). In comparison, the minimum value was observed at 100% BFCE (30.62 ± 1.3%) and complete supplementation by BFCE results in a decrease in the protein content by 36.19%.

##### Carbohydrates determination

**Fig. 8:**
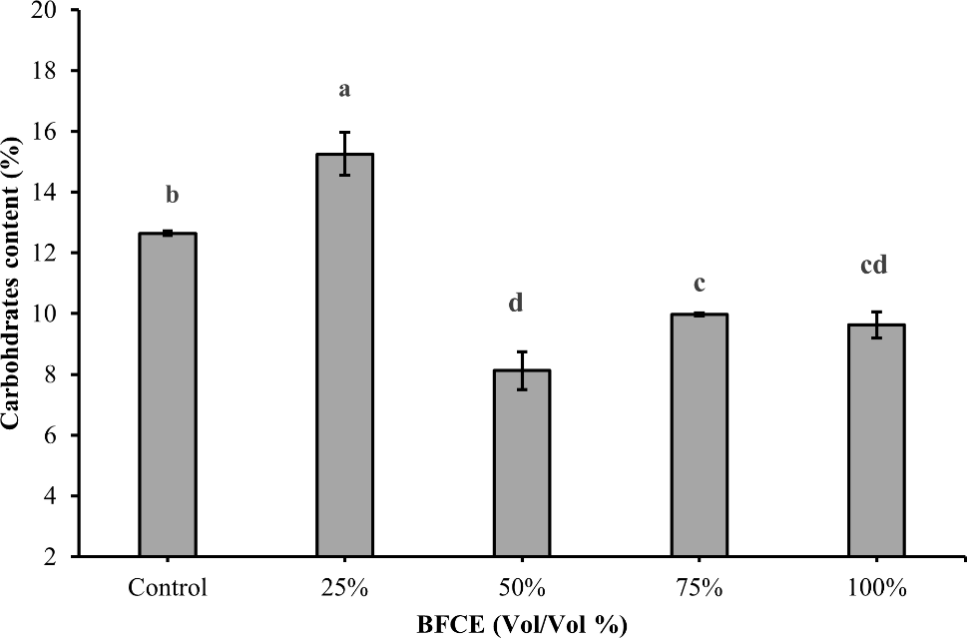
Carbohydrates content of *Leptolyngbya* sp. ± SE cultivated in the BG11 and BFCE treatments

The maximum carbohydrates content was observed at 25% BFCE (15.25 ± 0.7%), which increased by 20.7% over BG11. On the other hand, mixing between BFCE and BG11 medium in a 1:1 ratio caused a dramatically significant decrease in the carbohydrates contents. Moreover, the higher BFCE concentrations led to a significant decrease in the carbohydrate content as compared to the control medium (Fig. 8).

##### Lipid estimation

**Fig. 9:**
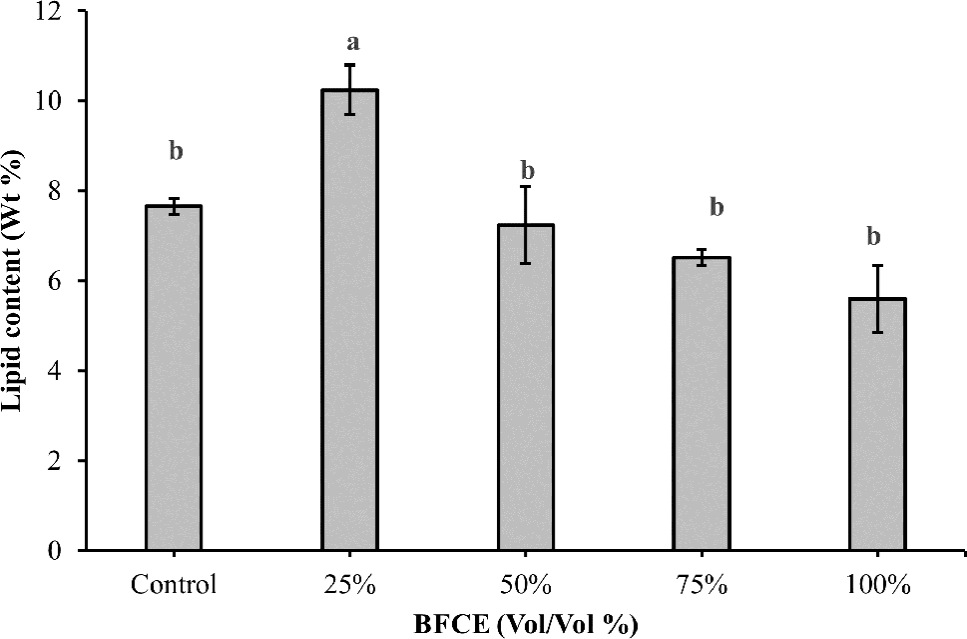
Lipid content of *Leptolyngbya* sp. ± SE cultivated in the BG11 and BFCE treatments

As shown in Fig. 9, the maximum lipid content was at 25% BFCE (10.23 ± 0.5), which was significantly increased by 33.72% (*p* ≤ 0.05) compared to BG11 (7.65 ± 0.1). However, the higher concentrations of BFCE showed a non-significant decrease compared to the control medium (BG11).

### Biosynthesis of selenium nanoparticles by crude phycocyanin extract

#### UV-Vis spectrophotometer

**Fig. 10:**
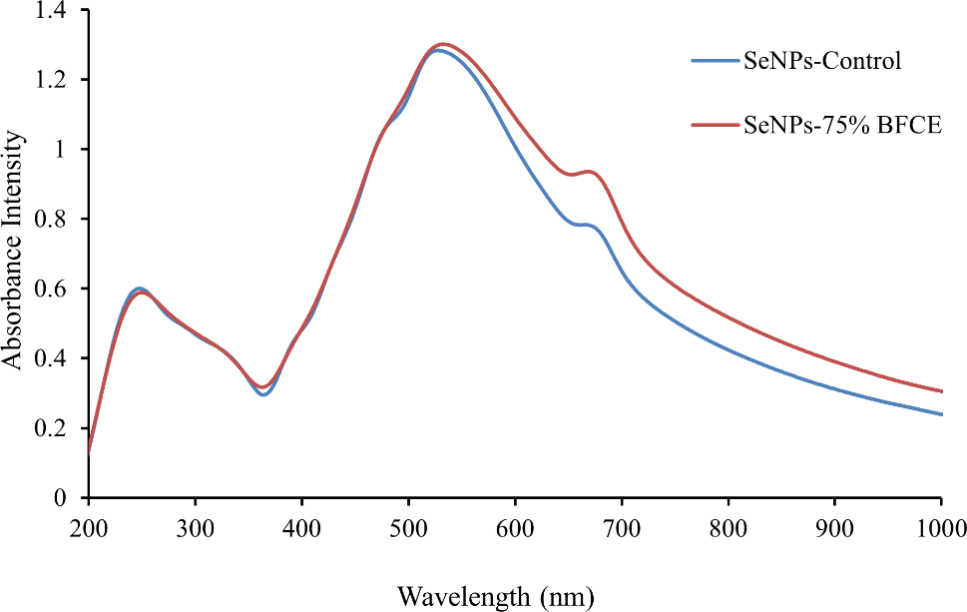
UV-Vis spectrophotometry analysis of Se-NPs synthesized from phycocyanin extract of *Leptolyngbya* sp

UV-Vis Spectrophotometry was conducted for the detection of synthesized Se-NPs from crude phycocyanin extract of both treatments which is displayed in Fig. 10. It was noticed that the synthesized Se-NPs from control (BG11) showed the maximum absorption peak at a wavelength of 528 nm at an absorbance of about 1.298. In comparison, the synthesized Se-NPs from 75% BFCE phycocyanin extract displayed a maximum absorption peak at the wavelength of 532 nm at an absorbance of about 1.301.

#### Transmission electron microscope analysis

**Fig. 11:**
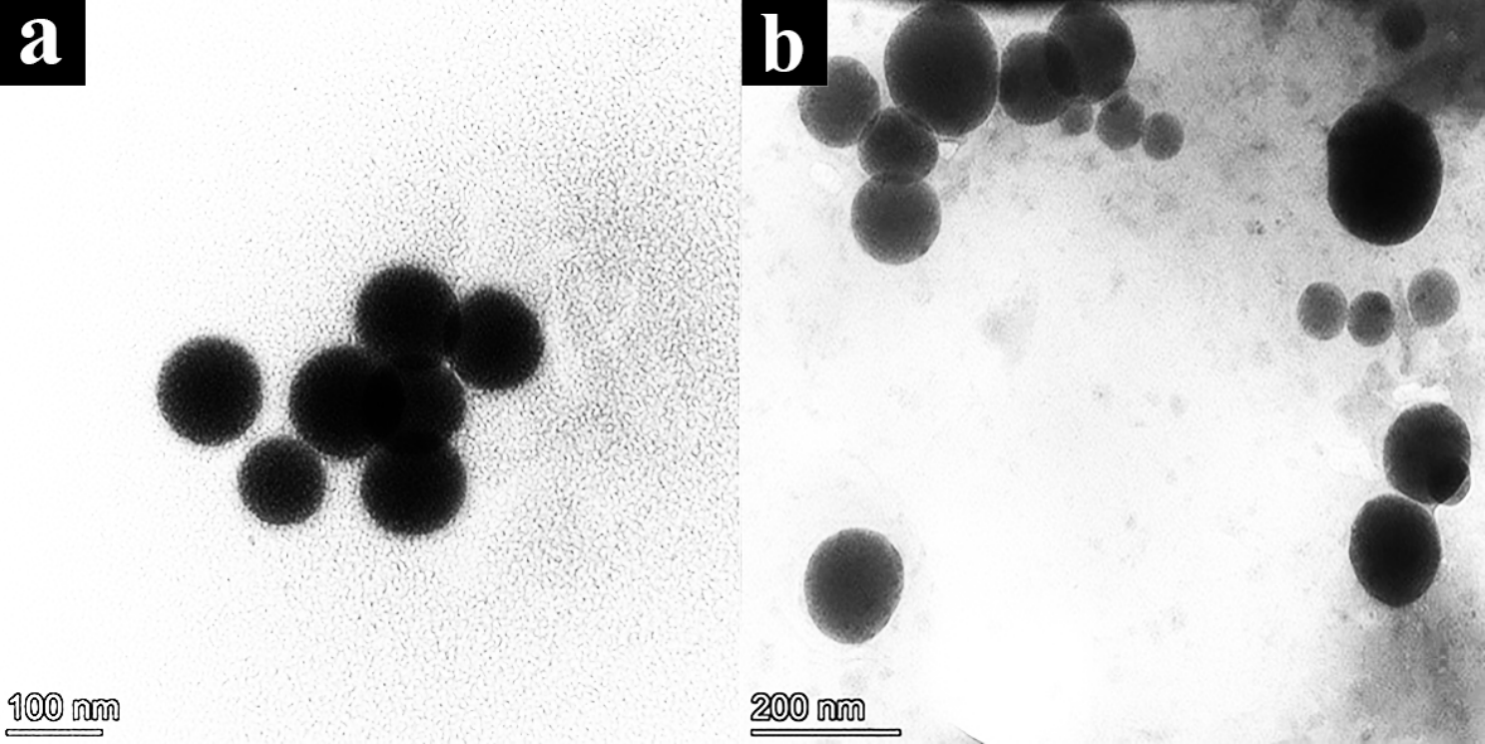
TEM images of SeNPs formed by crude phycocyanin extract of *Leptolyngbya* sp. (a) control (BG11) and (b) 75% BFCE

It was evident from the TEM investigation that the SeNPs had a spherical shape with a size of 44.45–209 nm with an average particle size of 95 nm in the control (BG11) medium (Fig. 11a). Likewise, the size of SeNPs formed from 75% BFCE crude phycocyanin extract ranged from 78 to 116 nm with a mean particle size of 96 nm (Fig. 11b).

#### X-ray diffraction (XRD)

**Fig. 12:**
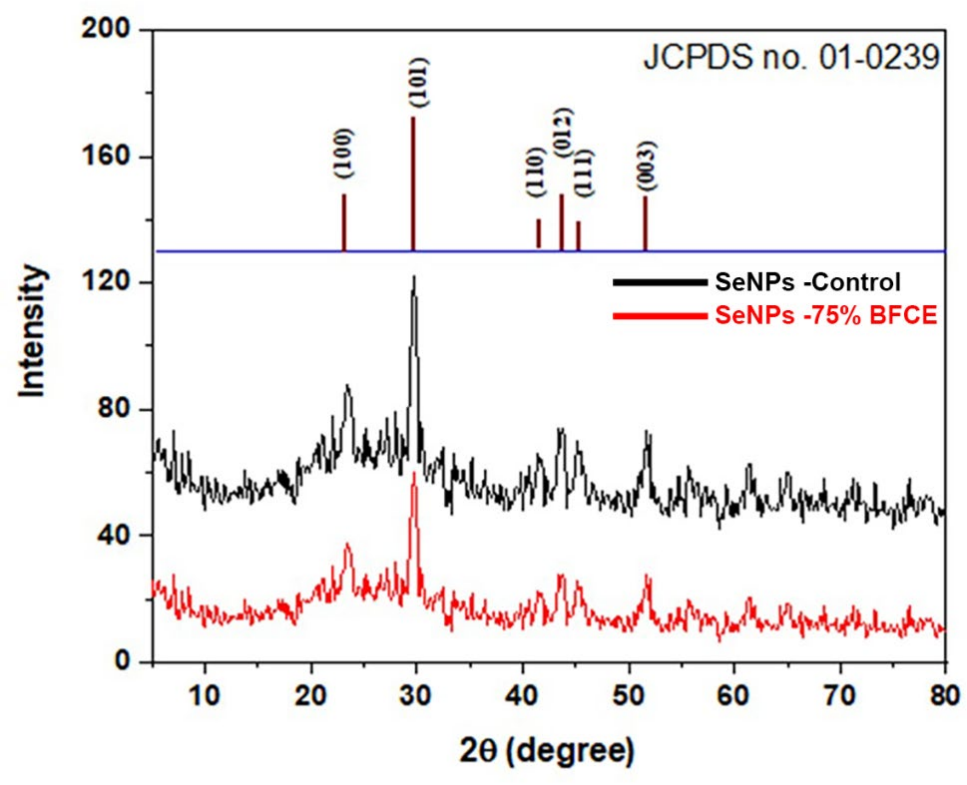
XRD analysis of Se-NPs synthesized from crude phycocyanin extract of *Leptolyngbya* sp. cultivated on control (BG11) and 75% BFCE

A typical XRD pattern of Se-NPs was displayed in Fig. 12. Six distinct, intense diffraction peaks were observed at 2θ = 23.36°, 29.76°, 41.44°, 43.84°, 45.21°, and 51.68°, corresponding to the (100), (101), (110), (012), (111), and (003) planes, which are characteristic of selenium nanoparticles when compared to the available standard selenium crystals, (JCPDS No. 01-0239). The more intense peak was at the (101) plane, indicating that the (101) plane was the primary orientation.

Moreover, the crystallite size was calculated from XRD data based on Scherrer’s equation using the most intense peak at about 29.76 for calculations. The estimated size was found to be 57.59 nm which is in agreement with that previously observed from the TEM image.

#### Zeta potential and polydispersity index (PDI) analyses

The prepared nanoparticles were immediately used after the preparation process for measuring the surface charge using the zeta potential technique and no evidence for agglomeration. It was found to be -17 mV and − 15.03 mV for the nanoparticles synthesized from the control (BG11) and 75% BFCE treatment, respectively. This negative charge on the surface of selenium nanoparticles will give it stability and resist its aggregation in the colloidal system. Moreover, the mean size of particles was found to be 335.9 nm with a PDI value of 0.1 and 464 nm with a PDI value of 0.31 for BG11 and 75% BFCE media, respectively. (Table 3)

**Table 3:** Zeta parameters of biosynthesized Se-NPs from both BG11 and BFCE treatment

Zeta parameters	Se-NPs-BG11	Se-NPs-75%BFCE
Zeta potential	-17mV	-15.03 mV
Zeta size	335.9 nm	464 nm
PDI	0.1	0.3

#### Fourier transform infrared (FTIR) spectroscopy

**Fig. 13:**
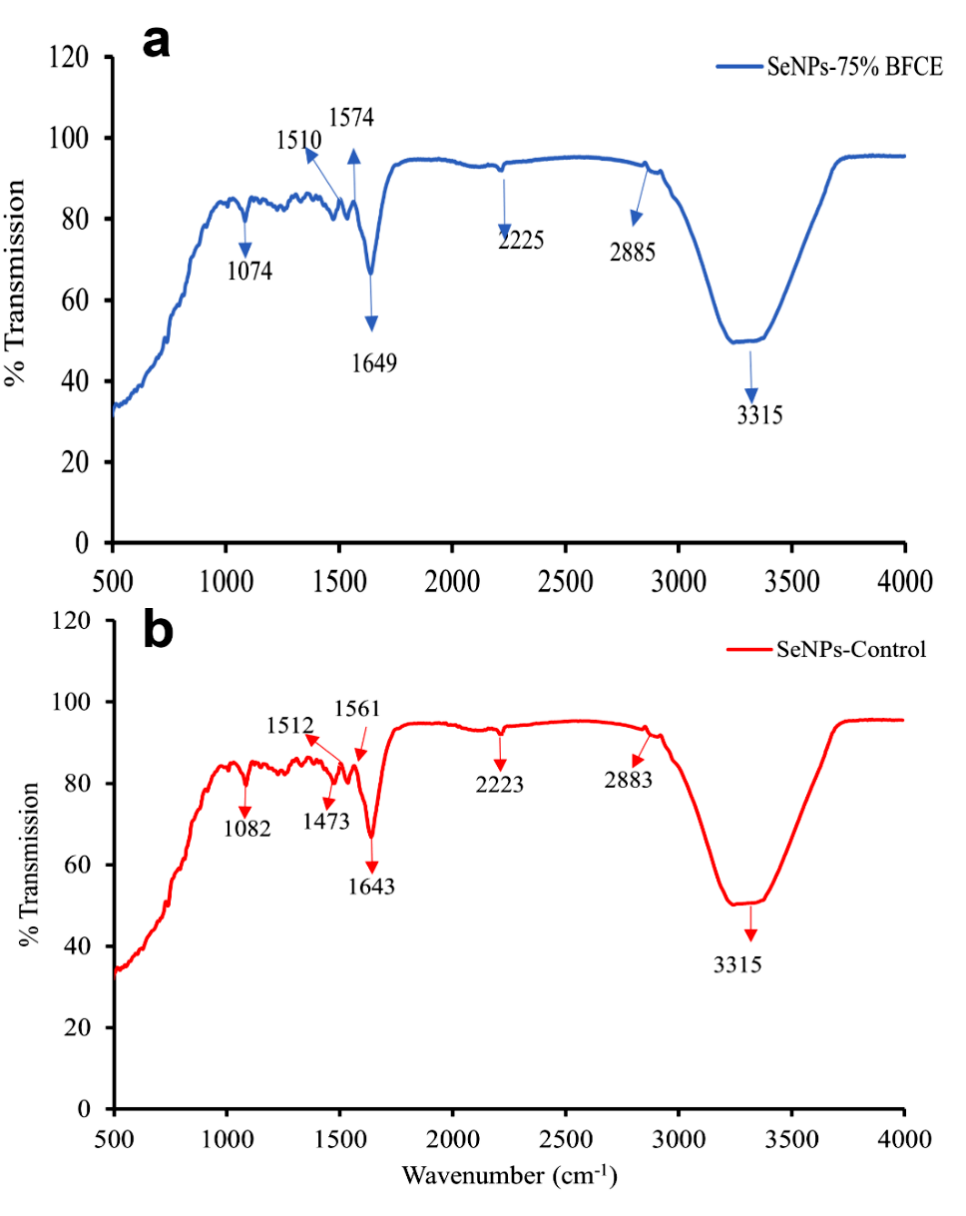
FTIR of biosynthesized Se-NPs from phycocyanin crude extract of **a**) 75% BFCE and **b**) BG11 medium

FTIR spectrum for Se-NPs synthesized from crude phycocyanin extract of *Leptolyngbya* sp. has absorption bands at 3315, 2883, 2223, 1643, 1561, 1512, 1473and 1082 cm^− 1^ for Se-NPs- control and approximately similar bands of 3315, 2885, 2225, 1649, 1575, 1510 and 1074 cm^− 1^in the spectrum of Se-NPs-75% BFCE (Fig. 13). The broad peak at 3315 cm^− 1^ is assigned to N–H (amino acids). The peak at 2883 can be attributed to stretching bands of CO of carboxylic anion. Moreover, a band at 1643 cm^− 1^ assigned to carbonyl and carboxylic (C-O) stretching bands of peptide linkages, and a peak found at 1561 cm^− 1^ represented carboxyl of carboxylic acid C-O-C.

### Application of biosynthesized selenium nanoparticles

The biosynthesized Se-NPs by the crude phycocyanin extract from *Leptolyngbya* sp, which was cultivated on both BG11 and 75% BFCE media, were examined for their biological activities against Gram-positive and Gram-negative bacteria, their antioxidant activity using DPPH free radicals in addition their cytotoxicity against breast cancer cell line, as following.

#### Antibacterial activity of biosynthesized Se-NPs

**Fig. 14:**
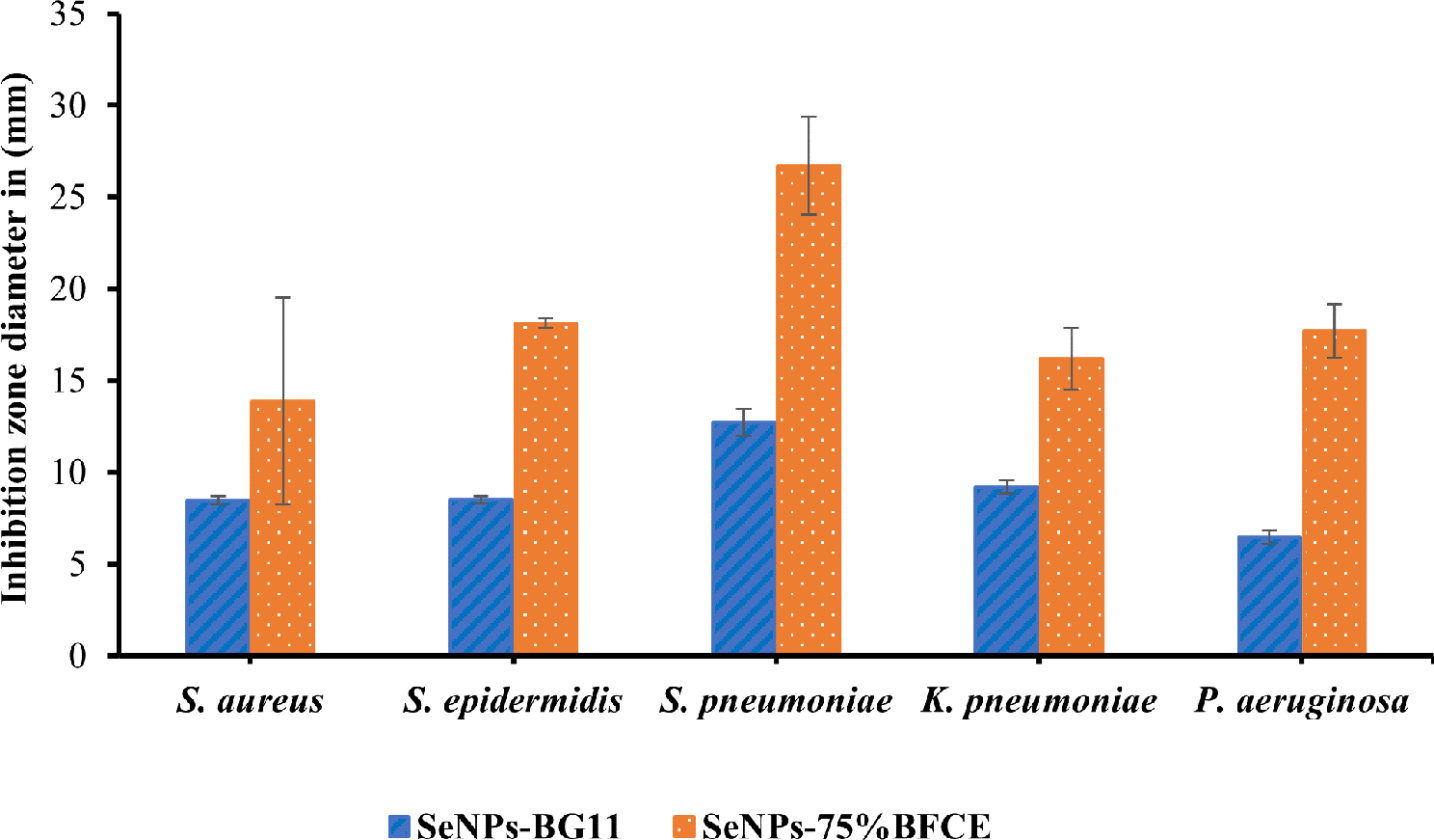
Antibacterial activity of biogenic Se-NPs from crude phycocyanin extract of *Leptolyngbya* sp extracted from BG11 (control) and 75% BFCE media

The antibacterial activity of biogenic Se-NPs from crude phycocyanin extract against three Gram-positive bacteria namely, *S. aureus*, *S. epidermidis*, and *S. pneumoniae*, and two Gram-negative bacteria namely, *P. aeruginosa*, and *K. pneumonia* was represented in Fig. 14. The synthesized Se-NPs from crude phycocyanin extract of 75% BFCE showed the highest antibacterial activity against all examined bacteria compared with Se-NPs from crude phycocyanin of BG11 treatment. Moreover, for SeNP-75% BFCE, the highest and lowest inhibition zones of 26.69 ± 2.6 mm and 13.8 ± 5 mm were against *S. pneumoniae* and *S. aureus*. Further, Se-NPs-BG11 showed the highest and lowest inhibition zones of 12.74 ± 0.73 mm and 6.455 ± 0.36 mm against *S. pneumoniae* and *P. aeruginosa*.

#### Anticancer activity of biosynthesized Se-NPs

**Fig. 15:**
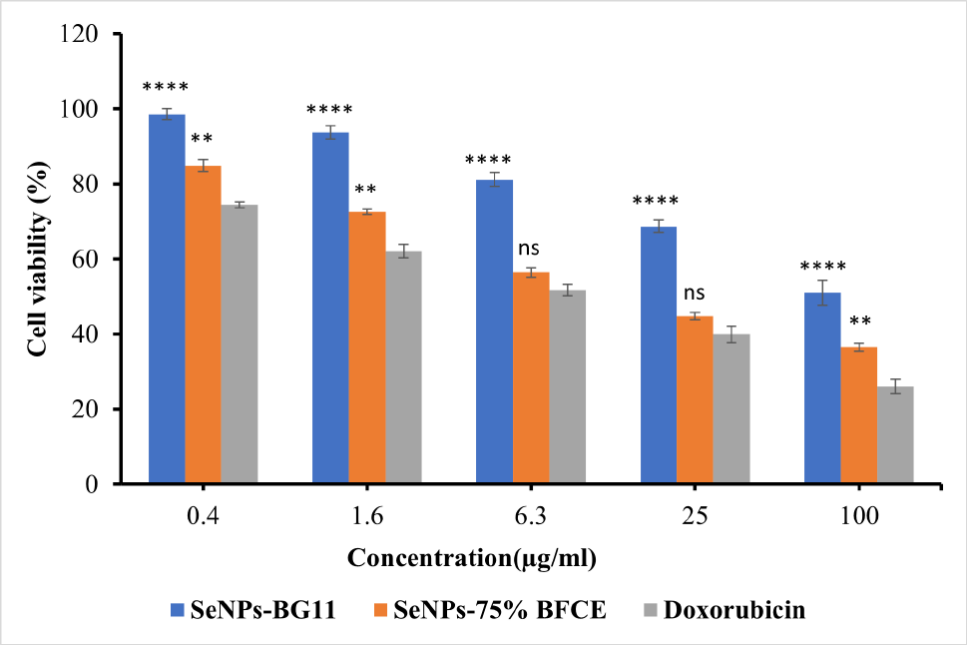
Effect of biogenic Se NPs and Doxorubicin as reference material on cell viability of MCF-7 cell line determined by MTT assay. Each value is represented as mean ± SD (*n* = 3, *p* ≤ 0.05). Two-way ANOVA with Tukey’s multiple comparisons test. Comparison with reference, ****p-level < 0.0001, **p-level < 0.01, and ns p-level > 0.05

The cytotoxic activity of the biogenic Se-NPs against the human breast cancer cell line (MCF-7) was investigated by MTT assay. As shown in Fig. 15, the cell viability of MCF-7 cell line decreased depending on Se-NPs concentrations. For Se-NPs from control treatment, the lowest concentration of 0.4 µg/ml showed the highest percentage of cell survival (98.567 ± 2.75%), while increasing the concentration to 100 µg/ml decreased the cell viability to 51.01 ± 1.43%, moreover, at all concentrations of Se-NPs-BG11 (0.4, 1.6, 6.3, 25, and 100 µg/ml), the cell viability (%) of MCF-7 was significantly higher than that of Doxorubicin at p-level < 0.0001. However, in Se-NPs-75% BFCE, concentrations of 0.4 and 100 µg/ml gave percentages of cell viability of 84.8 ± 1.071% and 36.5 ± 1.605%. In contrast, there was a non-significant difference (*p* ≤ 0.05) between Se-NPs-75% BFCE and Doxorubicin at concentrations of 6.3 and 25 µg/ml.

The inhibition concentration (IC_50_) of Se-NPs-BG11 and Se-NPs-75% BFCE were 132.58 ± 4.85 µg/ml and 17.31 ± 0.63 µg/ml, respectively and Doxorubicin was used as a reference anticancer agent with IC_50_ of 6.96 ± 0.26 µg/ml (Table 4).

**Table 4:** Cytotoxicity IC_50_ of Se-NPs synthesized from crude phycocyanin extract from *Leptolyngbya* sp. against human breast cancer cell line

Human cancer cell line	IC_50_ (µg/ml)		
	Se-NPs- BG11	Se-NPs- 75% BFCE	Doxorubicin (positive control)
MCF-7	132.58 ± 4.85	17.31 ± 0.63	6.96 ± 0.26

All results are expressed as mean ± SD from three experiments (*n* = 3)

#### Antioxidant activity of biosynthesized Se-NPs

The antioxidant activity of the biosynthesized Se-NPs was determined spectrophotometrically by 2,2-diphenyl-1-picrylhydrazyl (DPPH) assay using Torolox as a standard antioxidant. As shown in Table 5, the percentage of DPPH radical scavenging increased by increasing concentrations of Se-NPs. Moreover, Se-NPs-BG11 had the highest scavenging activity against DPPH ranging from 30.13 to 97.02% compared to that of Se-NPs-75% BFCE (18.23–95.96%) and Torolox (26.0- 97.40%) at the same concentrations. Additionally, The IC_50_ value was 60.58 µg/ml for the Se-NPs-BG11 which was lower than that of Torolox, while Se-NPs-75% BFCE had IC_50_ value of 134.8 µg/ml was higher than that of Torolox indicating that the Se-NPs-BG11 had higher antioxidant activity than that of Se-NPs-75%.

**Table 5:** DPPH radical scavenging activity (%) of the biosynthesized Se-NPs and the IC50 values

Concentrations of Se-NPs (µg/ml)	Se-NPs scavenging activity (%)		
	Se-NPs-BG11	Se-NPs-75% BFCE	Torolox
50	30.13	18.23	26.0
100	64.87	38.19	58.92
150	76.48	62.47	72.36
200	87.23	78.79	83.49
250	93.09	83.39	91.07
300	95.96	88.38	94.72
350	97.02	95.96	97.40
IC_50_	60.58 ± 0.7(µg/ml)	134.8 ± 1.5 (µg/ml)	85.72 ± 1 (µg/ml)

## Discussion

### Batch cultivation of *Leptolyngbya* sp. on BFCE as an alternative medium

In this study, BFCE was screened as a cheap feedstock for the cultivation of cyanobacterial isolate that was identified as *Leptolyngbya* sp. SSI24 with accession number PP723083. The photosynthetic activity, cell dry weight, photosynthetic pigments, and biochemical composition were estimated to investigate the influence of BFCE on the *Leptolyngbya* sp. growth performance. The Fv/Fm ratio of the dark-adapted microalgal and cyanobacterial cells is one of the chlorophyll fluorescence parameters that indicates the efficiency of photosynthesis and it is believed that a decrease in the Fv/Fm value gives an indication of environmental or nutritional stress [34]. However, the Fv/Fm ratio in most non-stressed plants and eukaryotic microalgae is 0.7–0.8, which higher than cyanobacterial cells depending on the strain and environmental conditions (0.3–0.4 or 0.4–0.6) [35–37]. These ranges are consistent with our results. The Fv/Fm ratio of control (BG11) and BFCE-supplemented media are almost the same values suggesting that the cyanobacterial cell is not stressed when grown on the BFCE media. Sometimes, it could show a low biomass with high-rate photosynthetic activity [38]. So, if all treatments, including the control, fluctuate within the Fv/Fm range of 0.30–0.50 still considered not stressed cells, which is confirmed by the non-significant changes in the biomass.

The difference in optical density of *Leptolyngbya* sp. SSI24 at BG11 medium was insignificantly compared to the BFCE-supplemented media, indicating that the high degree of adaptability of *Leptolyngbya* sp. SSI24 to the BFCE-supplemented media which was also coherent with the results of photosynthetic activity.

The cell dry weight reflects the growth of microalgal biomass, all tested BFCE concentrations stimulated the cell growth with the maximum biomass concentration obtained being 0.54 ± 0.047 g/l for the 100% BFCE culture The changes in the mode of cultivation of microalgae may influence the biochemical profile and biomass concentration [39]. Some microalgae and cyanobacteria can grow under mixotrophic conditions using inorganic and organic carbon sources achieving higher biomass yield than those of photoautotrophic, moreover, the mixotrophic cell obtains sufficient energy from metabolizing organic compounds and converts light energy into chemical energy through photosynthesis, which enhances nutrient uptake and helps the cells to reproduce thus improving the growth and biomass productivity [40]. In this study, BFCE obtained from the sugar beet industry was used as an alternative medium for the cultivation of *Leptolyngbya* sp. SSI24 which contains organic carbon sources, nitrogen, phosphorus, and some minerals [41]. The enhancement of cell dry weight in BFCE supplemented medium is due to the possibility of *Leptolyngbya* sp. SSI24 using BFCE for the mixotrophic cultivation. Similarly, an improvement in biomass concentration in *S. platensis* grown mixotrophically using molasses as an organic carbon source [42].

The chlorophyll content is crucial for autotrophic microalgae to maintain steady growth throughout photosynthesis [43]. It is known that cultivation conditions such as media composition, especially nitrogen and light intensity, may impact chlorophyll content [44]. In the current study, the chlorophyll-a content significantly decreased in BFCE-supplemented medium compared to the BG11 control medium. In the BG11 medium, the *Leptolyngbya* sp. SSI24 depends only on photosynthesis in its growth thus the production of chlorophyll-a increases, while in BFCE supplemented media the *Leptolyngbya* sp. SSI24 grows mixotrophically depending on the organic carbon source in the BFCE, which could lead to changes in photosynthetic apparatus thus the chlorophyll production decreases. The same findings were outlined by Caporgno MP, et al. [45], who demonstrated higher chlorophyll-a content in photo-autotrophy than in mixotrophy mode for *Chlorella protothecoides*. Moreover, decreasing the nitrogen content in the BFCE medium compared to BG11 may affect the photosynthetic pigment, according to Markou G, et al. [46], who documented that induction of nitrogen degradation enzymes and reduction of microalgal photosynthetic pigments were with nitrogen limitation.

Cyanobacteria are rich in water-soluble proteinaceous phycobiliproteins pigment with a spectrum ranging from 450 to 650 nm [47]. The effect of different BFCE supplementations on the phycocyanin production by *Leptolyngbya* sp. SSI24 was measured in mixotrophic and autotrophic conditions resulting in 25.29 ± 3.17, and 22.8 ± 1.27 mg/g, respectively. It deduced from this study that *Leptolyngbya* sp. SSI24 has an adaptable metabolism, as the mixotrophic and autotrophic cultures produced the phycocyanin in almost similar quantities. Also, Morais DV, et al. [48] obtained the same results when studying the effect of a synthetic medium supplemented with vinasse on the phycocyanin content of *Aphanothece microscopica*. Other studies, investigated the effect of acetate as an organic carbon source on the photosynthetic pigment in *S. platensis*, and they concluded that an acetate concentration of 4 g/l supported enhanced phycocyanin content compared to autotrophic [49].

The modifications in the composition of the nutrient medium may influence the composition of the produced biomass by altering the intracellular metabolites synthesis [50]. Nitrogen source is the most significant element affecting the cyanobacterial growth and the production of protein, nucleic acids, and pigments [51]. The protein content increases with abundant nitrogen supply, in contrast to the carbohydrate and lipid content, which are enhanced in the case of nitrogen limitation. These conditions induce the cell to produce more lipids and carbohydrates as energy storage components to lengthen the growth [52].

The maximum protein content was found in BG11 (47.9 ± 0.05%), while the lowest one was in 100% BFCE (30.62 ± 1.3%). This result may be attributed to the control medium (BG11) containing the optimal nitrogen concentration, which is used in protein biosynthesis in the cyanobacterial cell, while different BFCE concentrations contain a smaller amount of nitrogen compared to the control medium, so the protein content decreased. In a relevant study, *Lyngbya purpurem* was cultivated on BG11 (1.5 g/L NaNO_3_) and Bold 3 N medium (0.75 g/L NaNO_3_), and high protein content was observed in BG11 [53]. In agreement with Zarrinmehr MJ, et al. [54] observed that *Isochrysis galbana* protein content decreased with a decrease in nitrogen concentration.

The maximum carbohydrate and lipid contents were 15.25 ± 0.7 and 10.23 ± 0.5 at 25% BFCE. However, the higher concentrations have an adverse impact on carbohydrate and lipid content. As mentioned before, the decrease in the nitrogen content enhanced the production of these compounds, which are used as a storage material for growth. In 25% BFCE, the nitrogen content decreased by 25% compared to BG11 which maintains high carbonaceous storage molecules instead of nitrogenous molecules. This observation was in consistent with Ördög V, et al. [55] who mentioned that not only complete reduction in nitrogen content, but also median content of nitrogen resulted in higher biomass and lipid content. Moreover, mixotrophic cultivation supplies organic carbon source that could produce sufficient energy to enhance lipid synthesis [56].

### Biosynthesis of selenium nanoparticles by crude phycocyanin extract

Biosynthesis of Se-NPs was validated by UV-Vis Spectrophotometry, changing the blue color of phycocyanin to ruby red confirming the biosynthesis of Se-NPs by reduction of selenium ion in the solution. It was noticed two peaks, the first peak observed in the UV-region centered at 270 nm and may be assigned to organic molecules in reaction mixture such as protein [57, 58]. The second peak of each treatment was centered at 528 and 532 nm, respectively that could be attributed to surface plasmon resonance (SPR) of Se-NPs [59]. Generally, the size, shape, morphology, and composition of the prepared NPs influence the SPR band [60] Similarly, Alipour S, et al. [61] noticed the absorption peak of Se-NPs synthesized from *Spirulina platensis* was from 450 to 500 nm and Mosallam FM, et al. [62] who reported a UV absorption peak at 510 nm for Se-NPs produced using the aqueous extract of fermented Lupin (AEFL). Another study revealed that biosynthesized Se-NPs have an absorption peak at 379 nm [63]. Moreover, it was noticed that the two peaks were different which may be attributed to the distribution of Se-NPs, the first one can be observed from TEM to be ranged from 44.45 to 209 nm, and the second ranged from 78.71 to 116.86 nm.

TEM is an effective technique for determining the shape and size of nanoparticles. TEM images revealed that the synthesized Se-NPs were spherical in morphology and polydisperse with a mean size of 95 nm and 96.48 nm of crude phycocyanin extract from BG11 and 75% BFCE, respectively. In previous studies, the size of biosynthesized SeNPs was not consistent with our results, Alam H, et al. [64] whose synthesized spherical Se-NPs with a diameter in the range of 8–20 nm using an alcoholic extract of guava (*Psidium guajava)* leaf and biosynthesis of spherical SeNPs with a size range from 20 to 80 nm using the cell-free extract of *Gliocladium roseum* [58]. This variation in size can be explained by the variation in biomolecules present in an organism that manage the nanoparticles biosynthesis process [65].

X-ray diffraction pattern (XRD) analysis demonstrates whether the Se-NPs would possess an amorphous or crystalline structure [66]. Two sharp and strong reflection peaks were observed in the XRD pattern of Se-NPs at 23.36° and 29.76° at 2θ values which correspond to 100 and 101, respectively, which emphasizes the crystalline phase of the biosynthesized Se-NPs. These results were in conformity with other study [67]. On the other hand, some previous studies reported an amorphous structure of green synthesized Se-NPs [68, 69].

Evaluation of the long-term stability of nanoparticles in colloidal solution using the zeta potential that measures the surface charge of nanoparticles [70]. Nanoparticles with zeta potential value greater than + 30 mV or less than − 30 mV are considered to have sufficient repulsive force to prevent aggregation in the colloidal system. In this connection, the biosynthesized Se-NPs zeta potential equals − 17 mV and − 15.03 mV in the BG11 control and 75% BFCE treatment, respectively, indicating their stability. The negative values of zeta potential may be governed by the negatively charged functional groups adsorbed on the nanoparticle surface [71]. Additionally, the polydispersity index (PDI) value reveals the degree of homogeneity of the sample’s particles. Commonly, the PDI values are within a range of 0–1. The particles with lower PDI values close to zero are uniform and monodisperse-sized, while the higher PDI values close to one refer to a wide range of sizes (polydisperse) and tend to aggregate [72]. The PDI was calculated to be 0.1 and 0.3 for the crude phycocyanin extract of BG11 and 75% BFCE-made Se-NPs, respectively, demonstrating a uniform dispersion of Se-NPs in the suspension. Furthermore, the size obtained from the dynamic light scattering method (DLS) is higher than the actual size obtained by TEM, which may attributed to the DLS estimates the dynamic size of the Se-NPs that including a diameter of NPs and biomaterial adsorbed on its surface acting as stabilizers, but TEM measures only nano-crystal size without capping agent [16].

FTIR analysis was performed to recognize the potential biomolecules that manage the reduction of the Se^+^ ions and simultaneously cap the surface of selenium nanoparticles [73]. FTIR spectrum analysis of Se-NPs synthesized by crude phycocyanin extract of *Leptolyngbya* sp. SSI24 reveals various functional groups, which likely implied in the synthesis and stabilization of Se-NPs. A broad peak at 3315 cm^− 1^ may be assigned to N-H (amine group) of the protein, indicating the role of N-H containing proteins in the reduction of selenium ion forming Se-NPs, and a peak of 2883 cm^− 1^ could be assigned to C-H in methylene group of protein. Moreover, the peaks of 1512 and 1643 cm^− 1^ correspond to the amide II and I amide linkage of protein. Inconsistent with Jabs A [74], where the FTIR spectrum of protein showed two major bands known as amide I (1600–1700 cm^− 1^) and amide II. Amide I is the most intense band due to C = O stretching vibration, while amide II is due to the N-H bending vibration and C-N stretching vibration. The presence of peptides on the surface of selenium nanoparticles synthesized using phycocyanin extract plays several crucial roles. These peptides act as capping agents, providing steric stabilization to prevent agglomeration and control the size and shape of the nanoparticles during synthesis. They significantly enhance the biocompatibility of the nanoparticles, making them more suitable for biological applications. The peptide coating offers reactive groups for further functionalization, allowing for potential conjugation with other molecules. Some peptides may contribute additional antioxidant properties, complementing the antioxidant effects of selenium. Importantly, these surface peptides can facilitate targeted delivery to specific cells or tissues and enhance cellular uptake of the nanoparticles. The peptide layer also helps mitigate potential toxicity associated with bare selenium nanoparticles. Overall, the peptide coating derived from phycocyanin extract significantly improves the stability, functionality, and biological applicability of the synthesized selenium nanoparticles [75].

### Application of biosynthesized selenium nanoparticles

#### Antibacterial activity of Se-NPs

The evolution of antibiotic-resistant bacteria has increased the need to search for effective and safe alternative antimicrobial agents to replace antibiotics by using safe and effective green synthesized nanoparticles. In our study, the effectiveness of manufactured Se-NPs by crude phycocyanin extract of *Leptolyngbya* sp. SSI24 against some Gram-positive (*S. aureus*, *S. epidermidis*, and *S. pneumoniae*) and Gram-negative (*P. aeruginosa*, and *K. pneumonia*) was investigated. The effectiveness of Se-NPs from phycocyanin extract of 75% BFCE to inhibit the growth of both Gram-negative and Gram-positive bacteria was higher than that of Se-NPs from phycocyanin extract of BG11. This may be explained by nanoparticles size, the small size of Se-NPs enhances the internalization of nanoparticles. According to the results of TEM, the size of Se-NPs-75% BFCE was smaller than that of Se-NPs-control which improved the antimicrobial activity. In confirmatory with the study of Huang T, et al. [76], reported that the best antimicrobial activity against methicillin-sensitive and methicillin-resistant *S. aureus* was obtained using Se-NPs with an average diameter of 80 nm.

Moreover, the sensitivity of Gram-positive bacteria to biosynthesized Se-NPs was higher than Gram-negative bacteria, which may be assigned to the variation in the cell wall chemical composition between both types of bacteria [77]. Gram-negative bacteria contain a considerable amount of lipopolysaccharide, which is highly negative in nature, thus the repulsion force between the negatively charged Se-NPs and the lipopolysaccharide present in the membrane of Gram-negative bacteria increases, and consequently, the resistance toward Se-NPs increases. Conversely, Gram-positive bacteria contain a high amount of peptidoglycan and a limited amount of negatively charged lipopolysaccharide that decreases the repulsion force, and consequently, Se-NPs can be adhered on the surface of Gram-positive bacteria [78]. Arakha M, et al. [79] mentioned that the negatively charged NPs can significantly enhance the antibacterial activity and overcome the repulsion force between negatively Se-NPs and negatively charged bacteria by developing a mechanism known as molecular crowding. The nanoparticles inhibit bacteria by various mechanisms, inducing the formation of reactive oxygen species [80], changing the membrane permeability, and inhibiting the synthesis of proteins and DNA [81, 82]. Therefore, these results suggest that selenium nanoparticles may be used as an antibacterial agent alternative to antibiotics.

#### Anticancer activity of biosynthesized selenium nanoparticles

The MTT assay measures the cell viability as a function of the redox potential of cell and metabolically active cells convert MTT reagent to purple formazan crystals and its strength is directly proportional to viable cells. In this study, the MTT assay concluded that biosynthesized Se-NPs have inhibited the MFC7 cell viability depending on the concentration, meaning that increasing the concentration of Se-NPs resulted in decreasing the cell viability. Consequently, doxorubicin displayed potent cytotoxicity with an inhibition percentage of 73.95% in higher concentrations (100 µg/ml), followed by Se-NPs biosynthesized from phycocyanin of 75% BFCE (63.44%) and the lowest activity was recorded in Se-NPs biosynthesized from phycocyanin of BG11 (48.98%) at high concentration. Inhibition concentration (IC_50_) is the concentration that suppresses half of the tumor cell, so lower values of IC_50_ reveal a potent cytotoxic agent. According to Ioset J-R, et al. [83] who stated that the material with IC_50_ above 90 µg/ml has no effect on cell viability. In our study, Se-NPs synthesized by crude phycocyanin of 75% is strong cytotoxic agent with IC_50_ = 17.31 ± 0.63 µg/ml, while Se-NPs synthesized by crude phycocyanin extract of BG11has no cytotoxicity with IC_50_ of 132.58 ± 4.85 µg/ml, compared to the reference standard which has IC_50_ of 6.96 ± 0.26 µg/ml. In other studies, the inhibitory concentration (IC_50_) was found to be 50 µg/mL for Se-NPs synthesized from the crude extract of *Garcinia Mangostana* against Breast cancer cell line [84]. The cytotoxicity of Se-NPs may be explained by the fact that the negatively charged Se-NPs can be diffused through ion channels of cell membranes, and therefore, may interact with nitrogenous bases of DNA or intracellular proteins to cause cell cycle arrest, mitochondrial dysfunction, DNA fragmentation, and cell apoptosis [85, 86].

#### Antioxidant activity of biosynthesized Se-NPs

Antioxidant activity of biosynthesized Se-NPs was evaluated by using a stable free radical DPPH assay. This test depends on reduction of the free radical DPPH (purple color) into a non- radical stable DPPH-H (yellow color) by using antioxidant accompanied by a decrease in the absorbance at 517 nm [87]. The reduction of DPPH was inferred by a decrease in absorbance at 517 nm indicating higher antioxidant activity of DPPH scavenger [15]. In this study, the lower concentrations of Se-NPs had higher absorbance at 517 nm, while higher concentration showed disappearance of the DPPH color and consequently had lower absorbance values that results in higher DPPH scavenging activity.

The inhibition concentration that is necessary to inhibit half of cell viability is called IC_50_. The lowest IC_50_ values revealed the strongest antioxidant, in this study, Se-NPs synthesized from phycocyanin extracted from *Leptolyngbya* sp. SSI24 grown using BG11showed the lowest IC_50_ (60.58 ± 0.7 µg/ml) compared to Torolox used as standard antioxidant (85.7 ± 1 µg/ml), indicating that the high antioxidant activity of the formed Se-NPs-BG11. IC_50_ of Se-NPs formed in our study differed from that other literature. In a relevant study, Afzal B, Yasin D, Husain S, Zaki A, Srivastava P, Kumar R and Fatma T [10] mentioned that Se-NPs synthesized from *Arthrospira indica*,* Anabaena variabilis* NCCU-441, *Gloeocapsa gelatinosa* NCCU-430, *Oscillatoria* sp. NCCU-369, and *Phormidium* sp. NCCU-104 had IC_50_ values of 73.94 ± 1.53, 87.90 ± 1.34, 128.34 ± 2.77, 138.49 ± 0.81, and161.07 ± 0.46 µg/ml, respectively compared to ascorbic acid 55.74 ± 1.40 µg/ml. Moreover, Afzal B, Yasin D, Naaz H, Sami N, Zaki A, Rizvi MA, Kumar R, Srivastava P and Fatma T [31] synthesized extracellular Se-NPs from *Anabaena variabilis* that had antioxidant activity with IC_50_ value of 83.89 ± 2.11 µg/ml compared to ascorbic acid (56.36 ± 1.52 µg/ml.). In another study, Menon S, K.S SD, Agarwal H and Shanmugam VK [15] estimated the antioxidant activity of biosynthesized Se-NPs from ginger extract, and the Se-NPs had IC_50_ value of 125 µg/mL that is lower compared to ascorbic acid (250 µg/mL), revealing that Se-NPs is a potent antioxidant.

## Conclusion

The cyanobacterium *Leptolyngbya* sp. SSI24 was successfully cultivated in beet filter cake extract (BFCE) media as an alternative to the standard BG11 medium. Growth parameters like biomass, optical density, and photosynthetic efficiency were comparable between BG11 control and BFCE treatments. BFCE supplementation enhanced the biomass production of *Leptolyngbya* sp. by up to 42% compared to BG11 medium. This indicates BFCE can support mixotrophic growth. Photosynthetic pigments like chlorophyll were lower in BFCE media likely due to the availability of organic carbon. However, phycocyanin content was maintained at similar levels in BG11 and BFCE. Protein content decreased with increasing BFCE supplementation, while carbohydrates and lipids increased at 25% BFCE likely due to the lower nitrogen levels promoting carbo-storage compound accumulation. Crude phycocyanin extract from *Leptolyngbya* sp. cultivated in both BG11 and 75% BFCE successfully mediated the biosynthesis of spherical selenium nanoparticles (Se-NPs) with average sizes around 95–96 nm. XRD analysis confirmed the crystalline nature of the biosynthesized Se-NPs. The peaks at 23° and 29° 2θ correspond to the characteristic planes for selenium. The biosynthesized Se-NPs exhibited antibacterial activity against both Gram-positive and negative pathogens. Se-NPs from 75% BFCE phycocyanin showed higher potency. Se-NPs also displayed anticancer activity against the MCF-7 breast cancer cell line, with higher cytotoxicity for Se-NPs made using phycocyanin from *Leptolyngbya* sp grown using 75% BFCE.

Further, testing the ability of Se-NPs as free radical scavenger through scavenging DPPH free radical. The results showed that Se-NPs- BG11 had lower IC_50_ than that of Se-NPs-75% BFCE and Torolox.

## Data Availability

No datasets were generated or analysed during the current study.

## Abbreviations

BFC: Beet filter cake
BFCE: Beet filter cake extract
BSA: Bovine serum albumin
DLS: Dynamic light scattering
DPPH: 2,2-Diphenyl-1-picrylhydrazyl
FTIR: Fourier Transform Infra-red
Fv/Fm: Maximum photochemical efficiency
IC_50_: Half maximal survival inhibitory concentration
MCF-7: Human breast cancer cell line
MTT: 2-(4,5-dimethylthiazol-2-yl)-3,5-diphenyl-2 H-tetrazol-3-ium bromide)
PAM: Pluse- amplitude modulation
PC: Phycocyanin
PCR: Polymerase chain reaction
PDI: Polydispersity index
Se-NPs: Selenium nanoparticles
TEM: Transmission electron microscopy
XRD: X-ray diffraction
